# Schistosomiasis in Cattle in Corsica, France

**DOI:** 10.3201/eid2012.141474

**Published:** 2014-12

**Authors:** Didier Calavas, Paul M.V. Martin

**Affiliations:** Agence Nationale de Sécurité Sanitaire, Lyon Laboratory, Lyon, France

**Keywords:** schistosomiasis, humans, cattle, Corsica, France, trematodes, flukes, Schistosoma haematobium, S. bovis

**To the Editor:** The origin of the human cases of urinary schistosomiasis observed in France was recently identified (*1*,*2*). None of these patients had traveled to a disease-endemic area, but all had vacationed in Corsica and had swum in the Cavu River, near Porto-Vecchio in southern Corsica. The letter by Berry et al. to Emerging Infectious Diseases (*2*) reminded us that bovine schistosomiasis had been reported in Corsica in the past, up through the 1960s, in the same area.

In cattle, *Schistosoma bovis* has been found in Africa and the Middle East (Iraq, Israel), as well as in the Mediterranean Basin, especially in Sicily and Sardinia in Italy, and Corsica, France, where cases were reported as early as 1929 by Emile Brumpt (*3*,*4*). In addition, certain *Schistosoma* blood fluke species, especially *S. haematobium* and *S. bovis*, can share the same definitive hosts (humans or animals) and the same intermediate hosts, i.e., *Bulinus contortus* snails. Cattle, sheep and goats, horses, wild ruminants and rodents can all be definitive hosts of *S. bovis*.

In cattle, the clinical manifestations of infestation are poorly documented. In experimental animals, intermittent diarrhea has been observed, sometimes containing blood or mucus, in addition to a loss of appetite, progressive anemia, and, especially, blood eosinophilia, a sign which, as in humans, indicates that the infestation is recent. Under natural conditions, the disease is believed to be mainly subclinical and chronic. It should be noted that the acute form of the disease is more common in sheep (*5*). With regard to lesions, the disease is closer to intestinal schistosomiasis (caused by *S. mansoni*) than to urinary schistosomiasis. The lesions are characterized by the formation of gray-white granulomas ≥5 mmin diameter, or by polyps, and intestinal hemorrhaging due to bleeding of the granulomas formed during migration of the parasite’s eggs to the intestinal lumen. In the liver, granulomas may also be observed, as well as fibrosis of the portal vein. Hepatomegaly and cirrhosis may also be present. These lesions are caused by adult parasites in the mesenteric vessels and the portal vein.

The presence of *Bulinus truncatus contortus* (Michaud) (*Mollusca, Gastropoda, Hygrophila*) snails was mentioned as early as 1832, and the species was formally identified in 1922 in Corsica. Since that time it has been assumed that this mollusc could be a potential intermediate host for human (*6*) or bovine (*3*,*7*) schistosomiasis.

In 1963, Gretillat studied bovine schistosomiasis in Corsica (*8*). Investigations of *Bulinus* snails were conducted solely in the southern part of the island, in the area where Brumpt had described their presence 30 years earlier. *Bulinus* snails were identified in 4 rivers, the Rizzanese, Baraci, Ortolo and Spartano, especially in residual ponds of waterways sometimes quite close to the sea. At 2 sites, unidentifiable cercaria larvae were revealed through dissection (5 of 70 *Bulinus* snails in the Rizzanese, 26 of 50 in the Baraci). As part of the same study, slaughterhouse examination of 15 cattle from regions where *Bulinus* snails had been discovered revealed adult *Schistosoma* in the mesenteric and vesical veins, as well as in the liver and the portal system.

In a more comprehensive study (*9*), *Bulinus* snails were found in all of Corsica’s coastal rivers, except for those in the northwestern-most part of the island. However, of the 55 bodies of water where *Bulinus* snails were found, only 1 contained gastropods with *Schistosoma* cercariae, and results of a search for blood flukes in 220 small rodents (known for being susceptible to *S. bovis* and captured near bodies of water where *Bulinus* snails had been observed) were negative. 

We have found no other documentation on bovine schistosomiasis in Corsica between 1966 and the present time. Has this disease disappeared since the 1960s? Is it still present as an enzootic disease with silent transmission? It should be noted that the disease produces few or no clinical signs and that slaughterhouse detection requires dissection of the circulatory system of the abdominal cavity. In any case, the discovery of human cases of schistosomiasis proves that a human–*Bulinus* parasitic cycle exists in Corsica, and therefore an animal–*Bulinus* cycle may exist as well. For the sake of scientific interest, an investigation into the presence of *S. bovis* in ruminants in Corsica would be worthwhile. Moreover, the fact that both *Schistosoma* species use the same intermediate host, *Bulinus contortus* snails, could cause problems with differential diagnosis.
